# Genetic polymorphisms and clinical parameters associated with renal toxicity in Thai hematologic malignancy patients receiving high dose methotrexate

**DOI:** 10.1038/s41598-024-60334-w

**Published:** 2024-04-27

**Authors:** Palada Pitakkitnukun, Thanakit Pongpitakmetha, Thitima Benjachat Suttichet, Warumphon Sukkummee, Pajaree Chariyavilaskul, Chantana Polprasert

**Affiliations:** 1 Division of Hematology, Department of Medicine, Faculty of Medicine, Chulalongkorn University and King Chulalongkorn Memorial Hospital, Rama IV Road, Pathumwan, Bangkok, 10330 Thailand; 2 Department of Pharmacology, Faculty of Medicine, Chulalongkorn University, Rama IV Road, Pathumwan, Bangkok, 10330 Thailand; 3 Chula Neuroscience Center, King Chulalongkorn Memorial Hospital, Thai Red Cross Society, Bangkok, Thailand; 4 Center of Excellence in Clinical Pharmacokinetics and Pharmacogenomics, Faculty of Medicine, Chulalongkorn University, Bangkok, Thailand; 5 Center of Medical Diagnostic Laboratory, Faculty of Medicine, Chulalongkorn University, Bangkok, Thailand; 6 Center of Excellence in Translational Hematology, Faculty of Medicine, Chulalongkorn University, Bangkok, Thailand

**Keywords:** Haematological cancer, Haematological cancer, Predictive markers, Prognostic markers, Genetics research, Translational research, Pharmacogenetics

## Abstract

High-dose methotrexate (HD-MTX) is a widely used chemotherapy regimen for hematologic malignancies such as lymphomas and acute lymphoblastic leukemia, but its use can lead to adverse effects, including acute kidney injury (AKI), impaired liver function, and mucositis, causing extended hospital stays and delayed subsequent chemotherapy. Our study aimed to investigate the predictive factors for renal toxicities associated with HD-MTX in Thai patients undergoing treatment for hematologic malignancies. We enrolled 80 patients who underwent MTX-containing regimens, analyzing 132 chemotherapy cycles. The most common disease was primary central nervous system lymphoma (33%). Genetic polymorphisms were examined using the MassARRAY^®^ system, identifying 42 polymorphisms in 25 genes. Serum creatinine and MTX levels were measured 24 and 48 h after MTX administration. For the primary outcome, we found that the allele A of *MTRR* rs1801394 was significantly related to renal toxicity (odds ratio 2.084 (1.001–4.301), *p*-value 0.047). Patients who exceeded the MTX threshold levels at 24 h after the dose had a significantly higher risk of renal toxicity (OR (95%CI) = 6.818 (2.350–19.782), *p* < 0.001). Multivariate logistic regression analysis with a generalized estimated equation revealed hypertension and age as independent predictors of increased MTX levels at 24 h after the given dose.

## Introduction

Methotrexate (MTX) is an effective chemotherapy for the treatment of various hematologic malignancies, including primary central nervous system (CNS) lymphoma, diffuse large B-cell lymphoma, Burkitt lymphoma, extranodal NK/T-cell lymphoma, and acute lymphoblastic leukemia (ALL)^1,2^. As a folic acid antagonist, MTX inhibits the dihydrofolate reductase enzyme that catalyzes the conversion of dihydrofolate to tetrahydrofolate, an active form of folic acid that is crucial for the synthesis of deoxyribonucleic acid (DNA) and ribonucleic acid (RNA), resulting in the cytotoxic effect. When MTX is taken up into cells, it forms methotrexate-polyglutamate, which also inhibits DNA synthesis^3^.

Chemotherapy regimens for the treatment of hematologic malignancies generally contain high-dose MTX (HD-MTX; ≥500 mg/m^2^)^4^. However, MTX has a dose-dependent toxicity that affects multiple organ systems, including the kidneys, liver, mucosal tissue, and bone marrow. These adverse events lead to unwanted consequences, such as a prolonged duration of intravenous fluid hydration, the need to monitor kidney recovery, a prolonged duration of hospitalization, an increased risk of hospital-acquired infection, and delayed subsequent cycles of chemotherapy^4^.

Several previous studies focused on host genetic factors involved in the MTX metabolism pathway^5^. Example variants of *MTHFR C677T* (rs1801133), *ABCB1* C3435T (rs1045642), and *SLCO1B1* (rs11045879), and *MTRR* (rs1801394) were reported to be associated with MTX toxicity in hematologic malignancy conditions^2,6,7^. MTX is also used in autoimmune diseases. The findings of the role of *MTHFR C677T* (rs1801133) and *A1298C* in rheumatoid arthritis were associated with MTX toxicity in the Algerian population^8^. To our knowledge, the finalization of genetic variants that play a significant role in the prediction of MTX toxicities has not yet been concluded and warrants further study. Regarding adult Thai patients with hematologic malignancy receiving HD-MTX, this information is also scarce.

The primary objective of this study was to investigate the association between genetic variability in the MTX metabolism pathway and renal toxicity in Thai patients with hematologic malignancies. Secondary objectives were (1) evaluating the relationship between MTX levels at 24 hours after dosing and serum creatinine levels and (2) evaluating the relationship of MTX levels with other clinical data, for example, demographic data, diseases, chemotherapy regimens, the dose of MTX for each cycle, the laboratory index, the creatinine at baseline, and the creatinine after methotrexate administration.

## Methods

### Participants

This study was a single-center observational study at King Chulalongkorn Memorial Hospital (KCMH), Thai Red Cross Society, Bangkok, Thailand. The study was carried out in accordance with the Declaration of Helsinki and the Good Clinical Practice Guideline. The study was approved by the Institutional Review Board of the Faculty of Medicine, Chulalongkorn University (IRB No. 429/64, COA No. 733/2021). Informed consent was obtained from all participants and/or their legal guardian(s). For sample size calculation, we assumed from the most relevant study that reported the association of renal toxicity and *MTHFR* C677T polymorphisms on high-dose methotrexate-related toxicity in patients with primary CNS diffuse large B-cell lymphoma^9^. This study found that renal toxicity was 17% in wild genotype and 32% heterozygous/homozygous variant genotype. The total sample size of 278 (139 on each arm) for comparing the equality of two independent proportions with 80% power and a significance level of 0.05^10^. Despite the sample size calculation, only 80 participants were included due to resource and budget constraints. The inclusion criteria were Thai patients with hematologic malignancies older than 18 years and diagnosed with primary CNS lymphoma, diffuse large B-cell lymphoma, Burkitt lymphoma, extranodal NK/T-cell lymphoma, or ALL/lymphoblastic lymphoma (LBL). All participants were with a history of receiving a chemotherapy regimen containing intravenous HD-MTX (3–4 g/m^2^) with a creatinine clearance calculated by the Cockcroft-Gault equation of > 60 ml/min^11^ before starting HD-MTX from January 2015 to July 2022 at King Chulalongkorn Memorial Hospital. Patients with ascites or pleural effusions were excluded together with pregnant or breastfeeding patients. All participants received the standard HD-MTX protocol, which was adapted from previous studies^12–14^. The protocol included an intravenous mixture of 1,000 ml of 5% dextrose water together with 150 ml of 7.5% NaHCO_3_ and 20 meq of KCL administered at a rate of 250 ml/hr for 8 h before starting an intravenous HD-MTX. After starting an intravenous HD-MTX, the rate of the mixture of 5% dextrose water, 7.5% NaHCO3, and 20 meq of KCL was reduced to 150 ml/hr and continued for 24 h. Intravenous leucovorin was administered after 12 h of HD-MTX at a dose of 25 mg every 6 h for 12 doses or until the MTX level was < 0.1 μM (micromolar). The MTX level was monitored every 24 h after dosing until the MTX level was < 0.1 μM. Cessation of NSAIDs, PPIs, sulfamethoxazole and trimethoprim, penicillin, and aspirin-containing medications on the day of intravenous MTX administration and up to 72 h after the infusion initiation is mandatory.

### Genotyping

**Figure 1:**
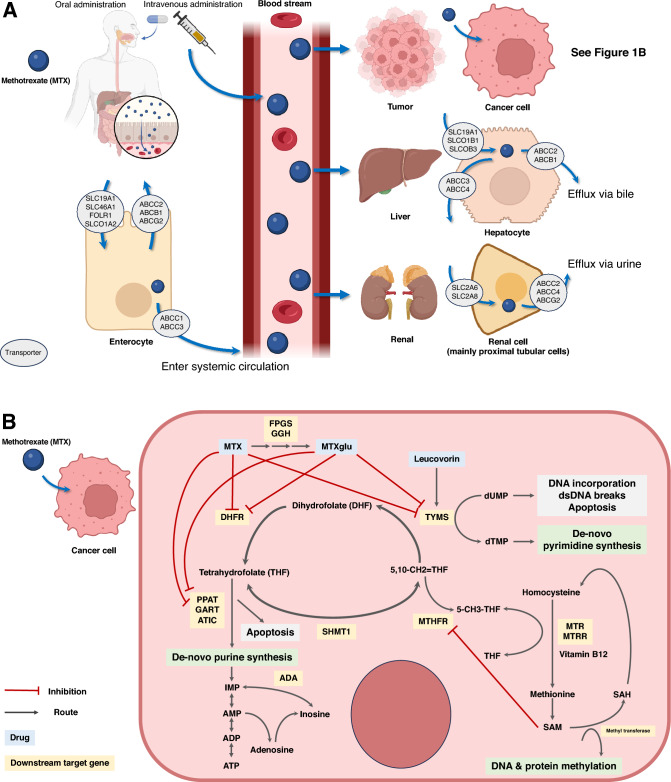
Methotrexate pathway and related pharmacogenomics. (**A**) This figure illustrates Methotrexate (MTX) pharmacokinetics, subject to genetic variations in membrane transporter proteins. MTX absorption in the gastrointestinal tract involves SLC19A1 and SLC46A1, while ABC transporters impact oral dosing bioavailability. Intravenous administration, prevalent in hematologic malignancy protocols, bypasses gastrointestinal transporters, ensuring heightened bioavailability. As kidneys are the primary excretory organs for MTX, the potential for kidney injury is influenced by genomic variations and patient-specific factors, underscoring the importance of understanding these intricacies in MTX pharmacokinetics. (**B**) This figure summarizes the complexity of the MTX pathway, purine synthesis, pyrimidine synthesis, folate metabolism, and related downstream target genes. These genomic variations resulted in the alteration of MTX level in the cellular level; thus, it might lead to clinical outcomes. MTX mainly inhibits *DHFR, TYMS, PPAT, GART,* and *ATIC* leading to cell apoptosis, especially in high turnover rate cells like cancer cells. The variant of *MTRR* gene allele rs1801394, which was significantly associated with acute kidney injury in this study, has its main action in changing from homocysteine to methionine. The effect of this variant in the alteration of cellular substrate has not been well-established to our understanding. This figure has been modified from the pharmacogenomics study from PharmGKB^®^^29,30^and created with BioRender.com. *MTX* methotrexate, *DHF* dihydrofolate, *THF* trihydrofolate, *IMP* inosine monophosphate, *AMP* adenosine monophosphate, *ADP* adenosine diphosphate, *ATP* adenosine triphosphate, *dUMP* deoxyuridine monophosphate, *dTMP* deoxythymidine monophosphate, *SAM* S-adenosyl methionine, *SAH* S-adenosyl homocysteine.

Whole blood was collected from each participant (3 ml) in an ethylenediaminetetraacetic acid (EDTA)-containing tube. Genomic DNA was extracted using a DNA mini kit (250) (QIAamp, Hilden, Germany) at the Advance Hematology Laboratory, Division of Hematology, Department of Medicine, Chulalongkorn University, Bangkok, Thailand. Forty-two polymorphisms of 25 genes were analyzed including *ARID5B* (rs10994982)*, ADORA2A* (rs5751876)*, ADORA3* (rs1544223*,* rs2298191, and rs3394), *ADA* (rs244076)*, ATIC* (rs12995526*,* rs16853834, rs16853826, rs2372536, rs4673990, rs4673993, and rs7563206), *BIRC5* (rs9904341)*, C1orf167* (rs1801131), *DDRGK1* (rs2295553)*, DHFR* (rs1643650 and rs7387), *FPGS* (rs1544105)*, GSK3B* (rs3732361)*, GGH* (rs11545078)*, HLA-E* (rs1264457)*, IL12B* (rs3212227)*, ITPA* (rs1127354)*, KLRC1* (rs2734414*,* rs2734440, and rs7301582), *MIR5189* (rs56292801)*, MROH2A* (rs10929303)*, MTHFD1* (rs2236225), *MTHFR* (rs1476413 and rs1801133)*, MTR* (rs1805087)*, MTRR* (rs162040 and rs1801394), *NR1I2* (rs3814055*,* rs6785049, and rs7643038), *PTPRM* (rs6506569)*,* and *TYMS* (rs2244500, rs2847153, and rs699517) by the MassARRAY^®^ system (Agena Bioscience, USA) at the Center of Excellence in Clinical Pharmacokinetics and Pharmacogenomics, Faculty of Medicine, Chulalongkorn University, Bangkok, Thailand. These genes were chosen from those that play important role in the MTX pathway and variants were found to pose some clinical significance^2,5–7,9^. The relationship between the MTX pathway and related pharmacogenomics is summarized in Fig. 1.

### Clinical and laboratory parameters

Comprehensive clinical data were retrospectively collected from the electronic medical record, including age, sex, body surface area (BSA; kg/m^2^), comorbidities, chemotherapy regimen, MTX dose, serum creatinine at baseline before MTX administration, serum creatinine at 24 h and 48 h after MTX administration, and MTX toxicity, including abnormal liver function test and mucositis.

The cut-off levels for MTX toxicities according to the 2016 National Protocol for the Treatment of Childhood Cancers and the Thai Pediatric Oncology Group (THAIPOG) protocol for ALL/LBL were applied^14^. Above MTX threshold levels were defined by serum concentrations of MTX ≥ 150 µM at 24 h after dose and > 0.4 µM at 48 h after dose for ALL/LBL^14^. For other diseases, levels above the MTX threshold were defined by serum MTX concentrations of ≥ 5 µM at 24 h after dose and > 0.4 µM at 48 h, which was adapted from the THAIPOG protocol^14^. Renal toxicity was defined as an increase in serum creatinine of > 25% from baseline at 24 and 48 h after the MTX dose for every disease. The definition for renal toxicity in this study was defined according to the THAIPOG protocol^14^ which had been adapted from the Children’s Oncology Group(COG) AALL0232^15^. The criteria for assessing changes in treatment for reducing MTX toxicity, particularly intravenous hydration and leucovorin administration within our protocol, were adjusted upon renal toxicity according to the criteria in the THAIPOG and COG protocols.

In the cases where MTX was administered in multiple cycles without renal toxicity, the data from the first cycle was used and considered as a ‘non-renal toxic group’ in the primary outcome analysis. For cases receiving multiple cycles and developing renal toxicity in any cycle, data from the first renal toxic cycle were used and analyzed as a ‘renal toxic group’.

For the secondary outcome, data was extracted from the first, third, and sixth MTX cycles. Associations between MTX levels at 24 h after dosing and serum creatinine levels were analyzed. The associations of MTX levels with other clinical parameters were also investigated. Liver toxicity was defined as an increase in aspartate aminotransferase (AST) or alanine aminotransferase (ALT) > 3.0 times the upper normal value; total bilirubin or direct bilirubin > 3.0 times the upper normal value^16^. Mucositis was classified using the Common Terminology Criteria for Adverse Events, version 5.0^16^. A mucositis score > 2 was considered to show MTX toxicity. Each chemotherapy cycle was considered as an independent event due to the need to eliminate MTX from circulation. In addition, the creatinine level needed to return to normal or show a tentative decrease for discharging patients.

### Statistical analysis

Statistical analysis was performed with IBM SPSS Version 29.0. Descriptive data were reported as frequencies and percentages. The mean and standard deviation (SD) were reported for the normally distributed data. For non-normally distributed data, median and interquartile ranges (IQR) were used. Visualized histograms and Shapiro-Wilk tests were used to test the normal distribution of variables. Categorical variables were analyzed using the Chi-square test, the odds ratio (OR) with a 95% confidence interval (95%CI). Univariate logistic regression and multivariate logistic regression with generalized estimating equations (GEE) for longitudinal data were used as appropriate. Statistical significance predictors from the univariate logistic regression at *p*-value < 0.05, <0.01, and <0.001 were selected into multivariate logistic regression with GEE models. The statistically significant level was *p*<0.05.

## Results

### Baseline characteristics and primary outcome

Eighty patients were enrolled with 132 total MTX cycles analyzed. Baseline characteristics are summarized in Table 1. The median±IQR age was 58.5±28.8 years. Female patients accounted for 61.3%. The most common hematologic malignancy was primary CNS lymphoma (n = 26, 32.5%). Different chemotherapy regimens were administered. The HD-MTX regimen was the most frequent (31%) followed by HD-MTX with Rituximab (16%). The three most common comorbidities were hypertension, dyslipidemia, and diabetes mellitus. The median dose of MTX was 3 g/m^2^. None of the ALL/LBL patients had a MTX level ≥150 µM at 24 hours after dosing. Among the rest of the subjects, 14 patients (17.5%) had an MTX level ≥5 µM at 24 hours after dosing. An increase in serum creatinine >25% from baseline was observed in 26 (32.5%) and 12 (15%) participants at 24 hours and 48 hours after MTX dosing, respectively. The genotype frequency of polymorphisms assessed in this study is shown in Supplementary Table 1. There was a significant risk of renal toxicity at 24 hours after MTX administration with *MTRR* rs1801394 allele A compared to variant allele G (OR (95%CI) = 2.084 (1.001–4.301), *p* = 0.047 (Table 2, Figure 1B). However, when data were analyzed as genotypes, no significant relationships were observed (Supplementary Table 2).

**Table 1:** Baseline characteristic of 80 patients.

Characteristics	Frequency (%)
Hematologic malignancy	
DLBCL/BL^+^ with CNS involvement	9 (10)
DLBCL with CNS prophylaxis	22 (28)
Extranodal NK/T-cell lymphoma	12 (15)
Acute lymphoblastic leukemia/lymphoma	11 (14)
Primary CNS lymphoma	26 (33)
Chemotherapy regimen	
High-dose MTX	25 (31)
High-dose MTX and rituximab	13 (16)
High-dose MTX-Ifosfamide	10 (13)
High-dose MTX-Ifosfamide and rituximab	8 (10)
Aspa/Met/Dex + /-BV^$^	12 (15)
Acute lymphoblastic leukemia induction protocol	10 (13)
Hyper-CVAD^&^	1(1.25)
CODOX-M/IVAC^#^	1(1.25)
Co-morbidities	
Atrial fibrillation/Myocardial ischemia	5 (5)
Diabetes mellitus	15 (18.8)
Dyslipidemia	17 (21.3)
HIV	3 (3.8)
Hypertension	22 (27.5)
Solid cancer	1 (1.3)
Recent/Past/Chronic viral hepatitis	14 (17.5)
Methotrexate status	
Median MTX dose (g/m^2^)	3.0
MTX dose 3 g/m^2^	44 (55)
MTX dose 3.5 g/m^2^	12 (15)
MTX dose 4.0 g/m^2^	24 (30)
24 h MTX ≥ 5 µM (exclude ALL/LBL)	14 (17.5)
48 h MTX levels > 0.4 µM	18 (22.5)
Median Cr baseline (mg/dl)	0.64 (IQR 0.3)
Cr rising > 25% (renal toxicity) at 24 h	26 (32.5)
Cr rising > 25% (renal toxicity) at 48 h	12 (15)

^+^Diffuse large B-cell lymphoma 9 cases, Burkitt lymphoma 1 case.

^$^Methotrexate, L-asparaginase, and Dexamethasone (+ − Brentuximab vedotin) for extranodal NK/T cell lymphoma.

^&^Hyperfractionated therapy containing cyclophosphamide, vincristine sulfate, doxorubicin.

hydrochloride (adriamycin), and dexamethasone.

^#^Cyclophosphamide, vincristine, doxorubicin, high-dose methotrexate/ifosfamide, etoposide, and high-dose cytarabine.

*MTX* methotrexate, *Cr* creatinine, *HIV* human immunodeficiency virus.

**Table 2:** Association between alleles and renal toxicity.

Gene	SNP	Event	Variant allele frequency (n(%))	Wild type allele frequency (n(%))	Odds ratio	95% confidence interval	*p*-value
*ATIC*	rs12995526	Renal toxic	40 (80)	10 (20)	0.835	0.357–1.956	0.678
		Non-renal toxic	91 (83)	19 (17)			
	rs16853834	Renal toxic	11 (23)	37 (77)	1.189	0.524–2.698	0.679
		Non-renal toxic	22 (20)	88 (80)			
	rs16853826	Renal toxic	22 (44)	28 (56)	1.272	0.646–2.506	0.487
		Non-renal toxic	42 (38)	68 (62)			
	rs2372536	Renal toxic	19 (38)	31 (62)	0.736	0.371–1.457	0.378
		Non-renal toxic	50 (45)	60 (55)			
	rs4673990	Renal toxic	40 (80)	10 (20)	0.835	0.357–1.956	0.678
		Non-renal toxic	91 (83)	19 (17)			
	rs4673993	Renal toxic	23 (46)	27 (54)	0.791	0.404–1.549	0.494
		Non-renal toxic	56 (52)	52 (48)			
	rs7563206	Renal toxic	10 (20)	40 (80)	1.125	0.483–2.620	0.785
		Non-renal toxic	20 (18)	90 (82)			
*DHFR*	rs1643650	Renal toxic	6 (12)	44 (88)	1.364	0.466–3.985	0.571
		Non-renal toxic	10 (9)	100 (91)			
	rs7387	Renal toxic	6 (13)	42 (88)	1.750	0.572–5.356	0.327
		Non-renal toxic	8 (8)	98 (92)			
*FPGS*	rs1544105	Renal toxic	33 (66)	17 (34)	0.762	0.372–1.561	0.457
		Non-renal toxic	79 (72)	31 (28)			
*GGH*	rs11545078	Renal toxic	7 (15)	41 (85)	0.892	0.346–2.299	0.812
		Non-renal toxic	18 (16)	94 (84)			
*MTHFD1*	rs2236225	Renal toxic	20 (40)	30 (60)	1.263	0.634–2.516	0.506
		Non-renal toxic	38 (35)	72 (65)			
*MTHFR*	rs1476413	Renal toxic	12 (24)	38 (76)	1.308	0.585–2.927	0.513
		Non-renal toxic	21 (19)	87 (81)			
	rs1801133	Renal toxic	6 (12)	44 (88)	0.801	0.294–2.187	0.665
		Non-renal toxic	16 (15)	94 (85)			
*MTRR*	rs162040	Renal toxic	34 (68)	16 (32)	1.077	0.528–2.199	0.839
		Non-renal toxic	73 (66)	37 (34)			
	rs1801394	Renal toxic	19 (38)	31 (62)	2.084	1.010–4.301	0.047*
		Non-renal toxic	25 (23)	85 (77)			

*p*-value: * < 0.05; ** < 0.01; *** < 0.001.

### Secondary outcome

Complete data from 132 MTX cycles used for the secondary analysis are presented in Supplementary Table 3. There was a significant association between exceeding the upper limit of MTX at 24 h after dosing and the risk of renal toxicity (OR (95%CI) = 6.818 (2.350–19.782), *p* < 0.001). In patients who encountered MTX toxicity, half of them received a lower dose of MTX in later cycles. Among those who received a reduced dose of MTX in subsequent cycles, all of them had no recurrent renal toxicity. Among those who maintained the same MTX dose, 40% experienced renal toxicity in the following cycles. Liver toxicity occurred in a total of 20 cycles (15%) of 17 patients (21.3%)*.* Mucositis was recorded in only 3 (2.3%) cycles in 3 patients (3.8%). Regarding the relationship between MTX levels at 24 h after dosing and clinical data, exceeding MTX levels at 24 h after dosing was significantly associated with age, HD-MTX and Rituximab regimen, diabetes mellitus, hypertension, dyslipidemia, and cardiovascular disease in the univariate logistic regression analysis. Having an MTX overdose that occurred in previous cycles was also associated with these events in subsequent cycles (Table 3). Parameters with significant associations from the univariate analysis (*p*-value < 0.05) were entered into the multivariate logistic regression analysis with GEE for the first round. However, this first model demonstrated that Hessian matrix singularity was observed and could not be used as an appropriate model. For the second round, we selected parameters with significant associations from the univariate analysis (*p*-value < 0.01) including age, essential hypertension, and prior MTX overdose in the previous cycle to analyze the multivariate logistic regression analysis with GEE. The second model demonstrated that the Hessian matrix was singular; hence, this model could not be applied. Thus, we selected parameters with significant associations from the univariate analysis (*p*-value < 0.001) including age and essential hypertension for the final model of the multivariate logistic regression analysis with GEE. The final model showed that hypertension (adjusted OR (95%CI) = 1.716 (0.556–2.875), *p* = 0.004) and age (adjusted OR (95%CI) =  − 0.070 (− 0.119– − 0.020), *p* = 0.006) were independent predictors of MTX overdose at 24 h after administration. (Table 3).

**Table 3:** Correlations between 24-h MTX level and clinical factors.

Variables		Univariate logistic regression–odds ratio (95% CI)	*P*-value	Multivariate logistic regression with generalized estimating equation–adjusted odds ratio (95% CI)	*P*-value
Age		1.09 (1.04–1.14)	< 0.001***	− 0.070 (− 0.119– − 0.020)	0.006**
Gender		1.73 (0.59–5.12)	0.320		
Body surface area (kg/m2)		0.76 (0.15–13.64)	0.760		
Chemotherapy regimens	High-dose MTX	2.73 (0.1–7.48)	0.050		
	High-dose MTX and Rituximab	3.57 (1.16–11.04)	0.030*		
	High-dose MTX-Ifosfamide	0.68 (0.18–2.53)	0.570		
	High-dose MTX-Ifosfamide and Rituximab	0.72 (0.15–3.42)	0.680		
	Aspa/Met/Dex + BV^$^	0.00	0.999		
	Aspa/Met/Dex	0.26 (0.03–2.04)	0.199		
	Acute lymphoblastic leukemia induction protocol	0.00	0.999		
	Hyper-CVAD^&^	0.00	1.000		
	CODOX-M/IVAC^#^	0.00	0.999		
Co-morbidities	Diabetes mellitus	3.01 (1.05–8.63)	0.040*		
	Hypertension	10.38 (3.62–29.77)	< 0.001***	1.716 (0.556–2.875)**	0.004**
	Dyslipidemia	3.23 (1.12–9.33)	0.030*		
	Chronic kidney disease	0.00	0.999		
	Solid cancer	0.00	0.999		
	Atrial fibrillation/Myocardial infarction	6.75 (1.53–29.71)	0.012*		
	HIV	0.00	0.999		
	Viral hepatitis	1.62 (0.48–5.49)	0.441		
Hematologic malignancy	Primary CNS lymphoma	2.32 (0.88–6.13)	0.090		
	Diffuse large B-cell lymphoma with CNS involvement	2.02 (0.50–8.22)	0.326		
	Diffuse large B-cell lymphoma with CNS prophylaxis	1.22 (0.37–4.07)	0.742		
	Burkitt lymphoma	0.00	0.999		
	Extranodal NK/T-cell lymphoma	0.20 (0.03–1.60)	0.130		
	Acute lymphoblastic leukemia	0.00	0.999		
	Lymphoblastic leukemia	0.00	1.000		
Side effect of MTX	Transaminitis	1.16 (0.38–3.50)	0.792		
	Mucositis	0.00	1.000		
MTX overdose in previous cycle		13.75 (2.33–81.25)	0.004**		

*p*-value: * < 0.05; ** < 0.01; *** < 0.001.

^$^Methotrexate, L-asparaginase, and Dexamethasone + Brentuximab vedotin for extranodal NK/T cell lymphoma.

^&^Hyperfractionated therapy containing cyclophosphamide, vincristine sulfate, doxorubicin.

hydrochloride (adriamycin), and dexamethasone, ^#^Cyclophosphamide, vincristine, doxorubicin, high-dose methotrexate/ifosfamide, etoposide, and high-dose cytarabine.

## Discussion

In our study, a total of 25 genes including 42 polymorphisms that relate to MTX metabolism were analysed by MassARRAY^®^ system. Given that previous reports used a wide range of methodologies, as well as different candidate genes or polymorphisms, the focus of our investigation was on candidate genes and polymorphisms that have been mentioned in earlier reports in this area^17,18^.

We found a potential association between wild-type allele A of *MTRR* rs1801394 and the risk of renal toxicity at 24 h after MTX dosing (OR (95% CI) = 2.084 (1.001–4.301); *p* = 0.047). In contrast, the previous reports showed conflicting results on the relationship between *MTRR* and MTX side effects, including renal toxicity^6,19^. The *MTRR* gene is responsible for maintaining optimal levels of methylcobalamin and activating cobalamin-dependent methionine synthase^17,18^. Variations in the *MTRR* gene are associated with a reduction in *MTRR* activity leading to dysregulation of folate metabolism and changes in homocysteine levels, which may influence the overall intracellular metabolism of MTX^6,7,20^. To date, the exact mechanism of *MTRR* gene affecting efficacy or toxicities of MTX treatment has not been fully investigated^19^. However, a previous study reported associations of calcineurin inhibitor-related nephrotoxicity to *MTRR* suggesting the role of *MTRR* SNPs altering the regulation of the homocysteine-methionine protein biosynthesis pathway affecting protein damage, cell death and immune activation^21^. A genetic study by Lievers et al.^20^ showed that *MTRR* 66A > G was related to an increasing risk of neural tube defect in pregnancy and cardiovascular disease. Another study on folate-related genes and MTX sensitivity in pediatric ALL reported that the *MTRR* 66A > G allele was less sensitive to MTX, which determined the efficacy of MTX in each individual^22^. In parallel with our study, a study conducted in pediatric ALL by Hoed et al.^23^ found that the wild-type *MTRR* was associated with higher MTX levels at 24 h. A study by Hao et al.^6^ showed that patients with the variant genotype *MTRR* rs1801394 (AG or GG) had a significant increase in serum MTX levels at 48 h and that change was not associated with renal toxicity from MTX which was discordance to our study. The population and method used in the study by Hao et al. were different from our study in the following ways: 1) enrolled only Chinese patients diagnosed with ALL, 2) different doses and leucovorin infusion protocol, and 3) different starting point of the monitoring time. In another study of Chinese patients by Lv et al.^19^, genetic variability in *MTRR* was not associated with adverse events from MTX. In this study, however, the patients were non-hematologic malignancies, and the dose of MTX was low (< 10 mg/week). The heterogeneity of the study population, different methodologies including drug dosage, drug monitoring period after MTX administration, cut-off point for renal toxicity, and MTX toxicity level, were all possible explanations for the inconsistent results.

Several publications widely studied *MTHFR* 677C > T showing that activity of the 677C > T allele reduces the MTHFR enzyme. However, the results were different between each study and most of the studies were unable to link the presence of these SNPs with an increased risk of MTX-mediated toxicity similar to our study^9,17^.

Our study showed the possible association between exceeding MTX levels at 24 h after dosing and the risk of renal toxicity. This was in line with the result of a study by Hao et al.^6^ in which a significant association was reported between renal toxicity and higher serum MTX concentrations at 24, 48, and 72 h and delay in MTX elimination^6^. A study by Miguel et al., also supported that there was an association between the MTX concentration at 24 h after dosing and the risk of renal toxicity^24^. More than 90% of MTX is excreted through the kidneys^4^. According to Yarlagadda et al., HD-MTX can cause renal toxicity due to precipitation of MTX crystals in the presence of acidic urine^25^. Obstructive uropathy in the renal tubules leads to acute renal tubular necrosis^26^. Intravenous hydration and urine alkalinization are the crucial steps to prevent MTX-related renal toxicity^26^. HD-MTX regimens in the many standard protocols, including our protocol, included urine alkalinization to ensure a urine pH of > 7.0 to prevent MTX-induced renal toxicity.

Our study also demonstrated that factors such as aging, diabetic mellitus, hypertension, cardiovascular disease, and dyslipidemia were associated with exceeding the 24-h MTX upper limit levels. Our proposed concept suggested that deterioration of the renal vascular system due to diabetic nephropathy, long-standing hypertension, dyslipidemia, or certain cardiovascular diseases could weaken renal function which led to more susceptible to renal injury from nephrotoxic drugs such as MTX. When methotrexate is introduced into patients with these co-morbidities, the kidneys may struggle to process and eliminate it effectively, increasing the risk of renal toxicity. In line with our study, Wight et al. observed that aging and hypoalbuminemia prolonged MTX clearance at 48 hours^27^. In contrast, Amitai et al. found that renal toxicity was associated with high lactate dehydrogenase and low albumin levels^28^. The proposed mechanism of hypoalbuminemia that elevates MTX levels was through the clinically significant third spacing of fluids^27^. These clinical factors and specific gene polymorphisms shall be considered when prescribing HD-MTX to patients when further consolidating studies are performed.

A key limitation of our study was the smaller number of patients than that had been calculated due to the limitation in resources and budget as previously mentioned. Another limitation was various treatment regimens might intervene with the renal function by other chemotherapy agents apart from the MTX itself. Lastly, in subsequent cycles, some patients had their MTX dosage reduced due to prior renal toxicity, which potentially complicated the interpretation of the results.

Future directions of MTX and gene polymorphism-directed treatment for risk-adapted renal toxicity in patients are an interesting area of research that needs additional clinical development leading to personalized treatment. Patients may undergo genetic testing to identify their specific genetic variations and polymorphisms that affect MTX metabolism and kidney function. This information might help tailor the dose and treatment regimens of MTX to minimize the risk of renal toxicity in the future. With a deeper understanding of genetics and drug response, algorithms and decision support tools can be developed to calculate optimal doses of MTX based on an individual’s genetic profile, age, and other risk factors.

## Conclusions

Among patients receiving HD-MTX, the *MTRR* allele variant rs1801394 wild-type allele A might potentially predict the association with renal toxicity. Hypertension and age were independent predictors of increased MTX levels at 24 h after the dose which were associated with renal toxicity.

### Supplementary Information

Supplementary Information.

## Data Availability

Raw data of patients’ demographic data and diseases with treatment such as chemotherapy regimens, MTX dosage of each cycle, the laboratory index; the creatinine at baseline, creatinine after methotrexate administration, and methotrexate levels at each time point were collected by PP. The genetic alleles and SNPs were kept by PP and TP. The data were provided within the manuscript or supplementary information files. Regarding drug (methotrexate) response with clinical significance from our study, the genetic polymorphisms data, *MTRR* allele variant rs1801394, were publicly deposited into the ClinVar under accession number SCV004217840.1 (https://www.ncbi.nlm.nih.gov/clinvar/variation/VCV000007029.20).
