# Supplementary material for: Genetic polymorphisms and clinical parameters associated with renal toxicity in Thai hematologic malignancy patients receiving high dose methotrexate
Source: Sci Rep. 2024 Apr 27;14:9695. doi: 10.1038/s41598-024-60334-w (PMC11055848; doi:10.1038/s41598-024-60334-w)
Supplement: Supplementary file 1 — Supplementary Information. [file 41598_2024_60334_MOESM1_ESM.docx]

**Supplementary data**

**Supplementary Table 1. Genotype frequency of genetic polymorphism of 25 genes.**

| ***Gene*** | **SNP** | **Genotype; Alleles** | **N (%)** |
| --- | --- | --- | --- |
| *MROH2A* | rs10929303 | Wild type; TT | 2 (2.5) |
|  |  | Homozygous variant; CC | 67 (87.0) |
|  |  | Heterozygous variant; TC | 8 (10.3) |
| *ARID5B* | rs10994982 | Wild type; AA | 21 (29.5) |
|  |  | Homozygous variant; GG | 23 (32.3) |
|  |  | Heterozygous variant; AG | 27 (38.0) |
| *ITPA* | rs1127354 | Wild type; CC | 46 (56.7) |
|  |  | Homozygous variant; AA | 27 (33.3) |
|  |  | Heterozygous variant; CA, CG | 7 (8.6), 1 (1.2) |
| *GGH* | rs11545078 | Wild type; GG | 59 (72.8) |
|  |  | Homozygous variant; AA | 3 (3.7) |
|  |  | Heterozygous variant; AG | 19 (23.4) |
| *HLA-E* | rs1264457 | Wild type; GG | 25 (30.8) |
|  |  | Homozygous variant; AA | 16 (19.7) |
|  |  | Heterozygous variant; AG | 40 (49.3) |
| *ATIC* | rs12995526 | Wild type; TT | 4 (4.9) |
|  |  | Homozygous variant; CC | 55 (67.9) |
|  |  | Heterozygous variant; CT | 22 (27.1) |
|  | rs16853834 | Wild type; CC | 56 (70) |
|  |  | Homozygous variant; TT | 11 (13.7) |
|  |  | Heterozygous variant; CT | 13 (16.2) |
|  | rs16853826 | Wild type; GG | 27 (33.3) |
|  |  | Homozygous variant; AA | 13 (14.8) |
|  |  | Heterozygous variant; GA | 42 (51.8) |
|  | rs2372536 | Wild type; CC | 25 (30.8) |
|  |  | Homozygous variant; GG | 12 (14.8) |
|  |  | Heterozygous variant; CG | 44 (54.3) |
|  | rs4673990 | Wild type; AA | 4 (4.9) |
|  |  | Homozygous variant; GG | 55 (67.9) |
|  |  | Heterozygous variant; GA | 22 (27.1) |
|  | rs4673993 | Wild type; TT | 27 (33.7) |
|  |  | Homozygous variant; CC | 25 (31.2) |
|  |  | Heterozygous variant; TC | 28 (35) |
|  | rs7563206 | Wild type; CC | 54 (66.6) |
|  |  | Homozygous variant; TT | 4 (4.9) |
|  |  | Heterozygous variant; CT | 23 (28.3) |
| *MTHFR* | rs1476413 | Wild type; CC | 59 (73.7) |
|  |  | Homozygous variant; TT | 13 (16.2) |
|  |  | Heterozygous variant; TC | 8 (10) |
|  | rs1801133 | Wild type; GG | 64 (79) |
|  |  | Homozygous variant; AA | 5 (6.1) |
|  |  | Heterozygous variant; GA | 12 (14.8) |
| *C1orf167* | rs1801131 | Wild type; TT | 45 (55.5) |
|  |  | Homozygous variant; GG | 10 (12.3) |
|  |  | Heterozygous variant; GT | 26 (32) |
| *FPGS* | rs1544105 | Wild type; CC | 9 (11.1) |
|  |  | Homozygous variant; TT | 40 (49.3) |
|  |  | Heterozygous variant; CT | 32 (39.5) |
| *ADORA3* | rs1544223 | Wild type; CC | 8 (9.8) |
|  |  | Homozygous variant; TT | 36 (44.4) |
|  |  | Heterozygous variant; TC | 37 (45.6) |
|  | rs2298191 | Wild type; TT | 27 (33.3) |
|  |  | Homozygous variant; CC | 12 (14.8) |
|  |  | Heterozygous variant; TC | 42 (51.8) |
|  | rs3394 | Wild type; TT | 12 (15) |
|  |  | Homozygous variant; CC | 40 (50) |
|  |  | Heterozygous variant; CT | 28 (35) |
| *MTRR* | rs162040 | Wild type; CC | 14 (17.2) |
|  |  | Homozygous variant; AA | 40 (49.3) |
|  |  | Heterozygous variant; CA | 27 (33.3) |
|  | rs1801394 | Wild type; AA | 41 (50.6) |
|  |  | Homozygous variant; GG | 6 (7.4) |
|  |  | Heterozygous variant; AG | 34 (41.9) |
| *MTR* | rs1805087 | Wild type; AA | 65 (80.2) |
|  |  | Homozygous variant; GG | 3 (3.7) |
|  |  | Heterozygous variant; AG | 13 (16.0) |
| *DHFR* | rs1643650 | Wild type; TT | 69 (85.1) |
|  |  | Homozygous variant; CC | 2 (2.4) |
|  |  | Heterozygous variant; CT | 10 (12.3) |
|  | rs7387 | Wild type; TT | 66 (84.6) |
|  |  | Homozygous variant; AA | 11 (14.1) |
|  |  | Heterozygous variant; AT | 1 (1.28) |
| *MTHFD1* | rs2236225 | Wild type; GG | 46 (56.7) |
|  |  | Homozygous variant; AA | 12 (14.8) |
|  |  | Heterozygous variant; GA | 29 (35.8) |
| *TYMS* | rs2244500 | Wild type; AA | 12 (14.8) |
|  |  | Homozygous variant; GG | 40 (49.3) |
|  |  | Heterozygous variant; GA | 29 (35.8) |
|  | rs2847153 | Wild type; GG | 36 (44.4) |
|  |  | Homozygous variant; AA | 17 (20.9) |
|  |  | Heterozygous variant; GA | 28 (34.5) |
|  | rs699517 | Wild type; CC | 11 (13.7) |
|  |  | Homozygous variant; TT | 60 (75) |
|  |  | Heterozygous variant; CT | 9 (11.2) |
| *DDRGK1* | rs2295553 | Wild type; CC | 16 (20.5) |
|  |  | Homozygous variant; TT | 51 (65,3) |
|  |  | Heterozygous variant; CT | 11 (14.1) |
| *ADA* | rs244076 | Wild type; TT | 57 (70.3) |
|  |  | Homozygous variant; CC | 6 (7.4) |
|  |  | Heterozygous variant; TC | 18 (22.2) |
| *KLRC1* | rs2734414 | Wild type; AA | 39 (48.7) |
|  |  | Homozygous variant; TT | 14 (17.5) |
|  |  | Heterozygous variant; TA | 27 (33.7) |
|  | rs2734440 | Wild type; CC | 15 (18.5) |
|  |  | Homozygous variant; TT | 29 (35.8) |
|  |  | Heterozygous variant; TC | 37 (45.6) |
|  | rs7301582 | Wild type; CC | 62 (79.4) |
|  |  | Homozygous variant; TT | 5 (6.4) |
|  |  | Heterozygous variant; TC | 11 (14.1) |
| *IL12B* | rs3212227 | Wild type; TT | 20 (24.6) |
|  |  | Homozygous variant; GG | 17 (20.9) |
|  |  | Heterozygous variant; TG | 44 (54.3) |
| *GSK3B* | rs3732361 | Wild type; AA | 26 (32) |
|  |  | Homozygous variant; GG | 13 (16) |
|  |  | Heterozygous variant; AG | 42 (51.8) |
| *NR1I2* | rs3814055 | Wild type; CC | 2 (2.5) |
|  |  | Homozygous variant; TT | 78 (97.5) |
|  |  | Heterozygous variant; CT | 0 |
|  | rs6785049 | Wild type; GG | 28 (37.3) |
|  |  | Homozygous variant; AA | 23 (30.6) |
|  |  | Heterozygous variant; AG, AT, GT | 22 (29.3), 1 (1.3), 1 (1.3) |
|  | rs7643038 | Wild type; GG | 5 (6.1) |
|  |  | Homozygous variant; AA | 54 (66.6) |
|  |  | Heterozygous variant; GA | 22 (27.1) |
| *MIR5189* | rs56292801 | Wild type; GG | 5 (6.4) |
|  |  | Homozygous variant; AA | 60 (76.9) |
|  |  | Heterozygous variant; GA | 13 (16.6) |
| *ADORA2A* | rs5751876 | Wild type; TT | 59 (80.8) |
|  |  | Homozygous variant; CC | 7 (9.5) |
|  |  | Heterozygous variant; CT | 7 (9.5) |
| *PTPRM* | rs6506569 | Wild type; TT | 27 (33.7) |
|  |  | Homozygous variant; CC | 18 (22.5) |
|  |  | Heterozygous variant; TC | 35 (43.7) |
| *BIRC5* | rs9904341 | Wild type; GG | 18 (24) |
|  |  | Homozygous variant; CC, AA, TT | 45 (60), 1 (1.3), 1 (1.3) |
|  |  | Heterozygous variant;  GT, CA, CG, CT, AG | 1(1.3), 3(4), 4(5.3) ,1(1.3),1 (1.3) |

**Supplementary Table 2. Correlations between genotype and renal toxicity at 24 hours**

| **Gene** | **Polymorphisms** | **Genotype** | **Total** | **No renal toxicity** | **Renal toxicity** | **P-value** |
| --- | --- | --- | --- | --- | --- | --- |
| *GGH* | rs11545078 |  | 80 (100) | 55 (68.8) | 25 (31.3) | 0.825 |
|  |  | Wild type; GG | 58 (72.5) | 41 (70.7) | 17 (29.3) |  |
|  |  | Homozygous variant; AA | 3 (3.8) | 3 (100) | 0 |  |
|  |  | Heterozygous variant; AG | 19 (23.8) | 12 (63.2) | 7 (36.8) |  |
| *ATIC* | rs12995526 |  | 80 (100) | 55 (68.8) | 25 (31.3) | 0.695 |
|  |  | Wild type; TT | 4 (5.0) | 2 (50) | 2 (50) |  |
|  |  | Homozygous variant; CC | 55 (68.8) | 38 (69.1) | 17 (30.9) |  |
|  |  | Heterozygous variant; CT | 21 (26.3) | 15 (71.4) | 6 (28.6) |  |
|  | rs16853834 |  | 80 (100) | 55 (69.6) | 24 (30.4) | 0.783 |
|  |  | Wild type; CC | 56 (70.0) | 40 (71.4) | 16 (28.6) |  |
|  |  | Homozygous variant; TT | 10 (12.5) | 7 (70) | 3 (30) |  |
|  |  | Heterozygous variant; CT | 13 (16.3) | 8 (61.5) | 5 (38.5) |  |
|  | rs16853826 |  | 80 (100) | 55 (68.8) | 25 (31.3) | 0.672 |
|  |  | Wild type; GG | 28 (35.0) | 21 (75) | 7 (25) |  |
|  |  | Homozygous variant; AA | 12 (15.0) | 8 (66.7) | 4 (33.3) |  |
|  |  | Heterozygous variant; GA | 40 (50.0) | 26 (65) | 14 (35) |  |
|  | rs2372536 |  | 80 (100) | 55 (68.8) | 25 (31.3) | 0.399 |
|  |  | Wild type; CC | 24 (30.0) | 16 (66.7) | 8 (33.3) |  |
|  |  | Homozygous variant; GG | 13 (16.3) | 11 (84.6) | 2 (15.4) |  |
|  |  | Heterozygous variant; CG | 43 (53.8) | 28 (65.1) | 15 (34.9) |  |
|  | rs4673990 |  | 80 (100) | 55 (68.8) | 25 (31.3) | 0.695 |
|  |  | Wild type; AA | 4 (5.0) | 2 (50) | 2 (50) |  |
|  |  | Homozygous variant; GG | 55 (68.8) | 38 (69.1) | 17 (30.9) |  |
|  |  | Heterozygous variant; GA | 21 (26.3) | 15 (71.4) | 6 (28.6) |  |
|  | rs4673993 |  | 79 (100) | 54 (68.4) | 25 (31.6) | 0.815 |
|  |  | Wild type; TT | 26 (32.5) | 17 (66.7) | 9 (33.3) |  |
|  |  | Homozygous variant; CC | 26 (32.5) | 19 (73.1) | 7 (26.9) |  |
|  |  | Heterozygous variant; TC | 27 (33.8) | 18 (66.7) | 9 (33.3) |  |
|  | rs7563206 |  | 80 (100) | 55 (68.8) | 25 (31.3) | 0.664 |
|  |  | Wild type; CC | 54 (67.5) | 37 (68.5) | 17 (31.5) |  |
|  |  | Homozygous variant; TT | 4 (5.0) | 2 (50) | 2 (50) |  |
|  |  | Heterozygous variant; CT | 22 (27.5) | 16 (72.7) | 6 (27.3) |  |
| *MTHFR* | rs1476413 |  | 79 (100) | 54 (68.4) | 25 (31.6) | 0.842 |
|  |  | Wild type; CC | 59 (73.8) | 41 (69.5) | 18 (30.5) |  |
|  |  | Homozygous variant; TT | 13 (16.3) | 8 (61.5) | 5 (38.5) |  |
|  |  | Heterozygous variant; TC | 7 (8.8) | 5 (71.4) | 2 (28.6) |  |
|  | rs1801133 |  | 80 (100) | 55 (68.8) | 25 (31.3) | 0.850 |
|  |  | Wild type; GG | 63 (78.8) | 43 (68.3) | 20 (31.7) |  |
|  |  | Homozygous variant; AA | 5 (6.3) | 4 (80) | 1 (20) |  |
|  |  | Heterozygous variant; GA | 12 (15.0) | 8 (66.7) | 4 (33.3) |  |
| \| *FPGS* \| \| --- \| \|  \| | \| rs1544105 \| \| --- \| \|  \| |  | 80 (100) | 55 (68.8) | 25 (31.3) | 0.759 |
|  |  | Wild type; CC | 8 (10.0) | 5 (62.5) | 3 (37.5) |  |
|  |  | Homozygous variant; TT | 40 (50.0) | 29 (72.5) | 11 (27.5) |  |
|  |  | Heterozygous variant; CT | 32 (40.0) | 21 (65.6) | 11 (34.4) |  |
| \| *MTRR* \| \| --- \| \|  \| | \| rs162040 \| \| --- \| \|  \| |  | 80 (100) | 55 (68.8) | 25 (31.3) | 0.972 |
|  |  | Wild type; CC | 14 (17.5) | 10 (71.4) | 4 (28.6) |  |
|  |  | Homozygous variant; AA | 41 (51.2) | 28 (68.3) | 13 (31.7) |  |
|  |  | Heterozygous variant; CA | 25 (31.3) | 17 (68) | 8 (32) |  |
|  | \| rs1801394 \| \| --- \| \|  \| |  | 80 (100) | 55 (68.8) | 25 (31.3) | 0.124 |
|  |  | Wild type; AA | 42 (52.5) | 33 (78.6) | 9 (21.4) |  |
|  |  | Homozygous variant; GG | 6 (7.5) | 3 (50) | 3 (50) |  |
|  |  | Heterozygous variant; AG | 32 (40.0) | 19 (59.4) | 13 (40.6) |  |
| \| *DHFR* \| \| --- \| \|  \| | \| rs1643650 \| \| --- \| \|  \| |  | 80 (100) | 55 (68.8) | 25 (31.3) | 0.809 |
|  |  | Wild type; TT | 67 (83.3) | 47 (70.1) | 20 (29.9) |  |
|  |  | Homozygous variant; CC | 3 (3.8) | 2 (66.7) | 1 (33.3) |  |
|  |  | Heterozygous variant; CT | 10 (12.5) | 6 (60) | 4 (40) |  |
|  | \| rs7387 \| \| --- \| \|  \| |  | 77 (100) | 53 (68.8) | 24 (31.2) | 0.317 |
|  |  | Wild type; TT | 64 (80.0) | 45 (70.3) | 19 (29.7) |  |
|  |  | Homozygous variant; AA | 1 (1.3) | 0 | 1 (100) |  |
|  |  | Heterozygous variant; AT | 12 (15.0) | 8 (70.3) | 4 (33.3) |  |
| \| *MTHFD1* \| \| --- \| \|  \| | \| rs2236225 \| \| --- \| \|  \| |  | 80 (100) | 55 (68.8) | 25 (31.3) | 0.595 |
|  |  | Wild type; GG | 46 (57.5) | 32 (69.6) | 14 (30.4) |  |
|  |  | Homozygous variant; AA | 24 (30.0) | 15 (62.5) | 9 (37.5) |  |
|  |  | Heterozygous variant; GA | 10 (12.5) | 8 (80) | 2 (20) |  |

**Supplementary Table 3. Chemotherapy cycles**

| **Chemotherapy regimen** | **Cycles (N)** |
| --- | --- |
| Adult-ALL protocol | 3 |
| AYA-ALL Induction protocol | 7 |
| High-dose MTX | 30 |
| High-dose MTX-Ifosfamide | 17 |
| High-dose MTX-Ifosfamide and Rituximab | 26 |
| High-dose MTX and Rituximab | 18 |
| Hyper-CVAD^&^ | 1 |
| Aspa/Met/Dex+BV^$^ | 7 |
| Aspa/Met/Dex | 20 |
| CODOX-M/IVAC^#^ | 3 |

Adult-ALL = Adult-acute lymphoblastic leukemia protocol

AYA-ALL = Adolescents and young adults with an acute lymphoblastic leukemia

^&^Hyperfractionated therapy containing cyclophosphamide, vincristine sulfate, doxorubicin

hydrochloride (adriamycin), and dexamethasone

^$^Methotrexate, L-asparaginase, and Dexamethasone +Brentuximab vedotin for extranodal NK/T cell lymphoma

^#^Cyclophosphamide, vincristine, doxorubicin, high-dose methotrexate/ifosfamide, etoposide, and high-dose cytarabine
